# How Multidimensional Is Emotional Intelligence? Bifactor Modeling of Global and Broad Emotional Abilities of the Geneva Emotional Competence Test

**DOI:** 10.3390/jintelligence9010014

**Published:** 2021-03-05

**Authors:** Daniel V. Simonet, Katherine E. Miller, Kevin L. Askew, Kenneth E. Sumner, Marcello Mortillaro, Katja Schlegel

**Affiliations:** 1 Department of Psychology, Montclair State University, Montclair, NJ 07043, USA; askewk@montclair.edu (K.L.A.); sumnerk@montclair.edu (K.E.S.); 2 Mental Illness Research, Education and Clinical Center, Corporal Michael J. Crescenz VA Medical Center, Philadelphia, PA 19104, USA; katherine.miller13@va.gov; 3 Swiss Center for Affective Sciences, University of Geneva, 1205 Geneva, Switzerland; marcello.mortillaro@unige.ch; 4 Institute of Psychology, University of Bern, 3012 Bern, Switzerland; katja.schlegel@psy.unibe.ch

**Keywords:** emotional intelligence, Geneva Emotional Competence Test (GECo), Cattell-Horn-Carroll (CHC) theory, multidimensionality, S-1 Bifactor Modeling

## Abstract

Drawing upon multidimensional theories of intelligence, the current paper evaluates if the Geneva Emotional Competence Test (GECo) fits within a higher-order intelligence space and if emotional intelligence (EI) branches predict distinct criteria related to adjustment and motivation. Using a combination of classical and S-1 bifactor models, we find that (a) a first-order oblique and bifactor model provide excellent and comparably fitting representation of an EI structure with self-regulatory skills operating independent of general ability, (b) residualized EI abilities uniquely predict criteria over general cognitive ability as referenced by fluid intelligence, and (c) emotion recognition and regulation incrementally predict grade point average (GPA) and affective engagement in opposing directions, after controlling for fluid general ability and the Big Five personality traits. Results are qualified by psychometric analyses suggesting only emotion regulation has enough determinacy and reliable variance beyond a general ability factor to be treated as a manifest score in analyses and interpretation. Findings call for renewed, albeit tempered, research on EI as a multidimensional intelligence and highlight the need for refined assessment of emotional perception, understanding, and management to allow focused analyses of different EI abilities.

## 1. Introduction

Emotional intelligence (EI) is viewed as a capacity to understand how emotions differ, to grasp similarities and distinctions between emotive signals, to formulate general rules about effective regulatory strategies, and to understand when those rules do not apply. One motivating interest in EI is the notion that life success requires more than analytical and technical reasoning (Ybarra et al. 2014). However, Goleman’s (1995) early claim that EI was more important for success than cognitive ability led scholars to raise concerns about its conceptual underpinnings (Locke 2005), predictive utility (Antonakis 2004), and logical basis for dictating a “correct” way to emotionally respond in any given situation (Brody 2004). Such critiques questioned whether and under what conditions EI can be considered a valuable addition to existing individual difference taxonomies and, more generally, how the parameters of psychometric and process models of EI test data should be interpreted with reference to research on cognition and emotion. Recent meta-analyses (Joseph and Newman 2010), measurement development (Schlegel and Mortillaro 2019), and focused investigations (Parke et al. 2015) have stimulated a tempered revival of matured EI frameworks, which assimilate intelligence research and emotional mechanisms (Mestre et al. 2016). The current study builds upon these advances by using the recently developed Geneva Emotional Competence Test (GECo) (Schlegel and Mortillaro 2019) to model the structure of ability-based EI and identify the unique contribution of separate EI branches on multiple emotion-centric student outcomes.

### 1.1. Measuring Emotional Intelligence

The predominant framework for understanding EI is Mayer and Salovey’s (1997) four-branch hierarchical model, which delineates EI into four broad abilities (or branches) increasing in cognitive complexity. The most rudimentary skill is accurately recognizing emotional signals (perception), which allows one to integrate emotional information into thinking (facilitation) and to build knowledge about the nature of emotional experiences (understanding). Understanding then informs strategic reasoning about how to modify emotional states to attain personal aims (regulation). However, the MSCEIT facilitation branch is empirically and conceptually redundant with other branches (Fan et al. 2010), leading scholars to focus on a simplified three-branch model comprised of emotional perception, understanding, and regulation (Joseph and Newman 2010; MacCann et al. 2014).

A major limitation of EI research is potential mono-method bias due to overreliance on the *Mayer-Salovey-Caruso Emotional Intelligence Test* (MSCEIT) (Mayer et al. 2003) as the primary tool for operationalizing EI. This is problematic as the legitimacy of an entire field rests predominantly on findings from a single instrument. Further, there are numerous challenges to the MSCEIT’s construct validity (e.g., Brody 2004; Fiori and Antonakis 2011; Maul 2012). These issues include items which do not capture intelligent responses to emotional situations (Maul 2012), psychometric issues with structural fidelity, reliability, validity, and item difficulty (Fiori et al. 2014; Keele and Bell 2008; Zeidner and Olnick-Shemesh 2010), overlap with personality (Fiori and Antonakis 2011), and a scoring procedure that relies on a consensus as opposed to performance (MacCann et al. 2004).

To address these limitations, Schlegel and Mortillaro (2019) developed the Geneva Emotional Competence Test (GECo), a theoretically grounded ability-EI battery for public research use. Contextualized to the workplace, the GECo draws from the three-branch model but expands the breadth and difficulty of items to more comprehensively elicit emotional abilities and develops maximal scoring items using research informed findings on emotions. For emotional perception (also labeled recognition), rather than presenting static images and non-emotive objects as item stimuli, the GECo uses dynamic, multi-media video displays of facial, acoustic, and body cues to capture test-takers’ skill in discriminating distinct emotional states. Based on emotional appraisal theory, the emotion understanding test uses objectively correct responses determined by which emotions are produced by prototypical situational profiles, such as differentiating mild irritation from intense anger (MacCann and Roberts 2008; Roseman 2001). Finally, the GECo divides the regulatory branch into emotion management in self (hereafter referred to as emotion regulation) and others (hereafter referred to as emotion management). Emotion regulation captures the use of adaptive cognitive strategies to modify internal emotional states (e.g., reappraisal, suppression) and emotion management is the use of behavioral strategies to successfully manage others’ emotions (e.g., accommodation, compromise). Both regulatory branches are scored according to empirical findings on the most effective cognitive strategies for mitigating negative emotions (Garnefski et al. 2001) and the most effective tactics for influencing others in mixed-motive situations characterized by diverse pressures, power dynamics, and goals (Thomas 1976).

To date, only a few investigations have examined the GECo’s psychometric properties by testing oblique intelligence solutions, evaluating item precision, and comparing criterion validity in relation to the MSCEIT (Schlegel and Mortillaro 2019; Völker 2020). However, research has yet to model a bifactor or hierarchical structure, evaluate if broad versus specific GECo abilities predict different criteria above personality, or determine if the branches produce univocal and reliable sub-scale scores.

### 1.2. Is Emotional Intelligence Unidimensional?

EI is viewed as a “broad” intelligence involved in emotional reasoning, but the breadth of this capability is debated (MacCann et al. 2014). Questions remain whether EI should be predominantly understood as a single latent factor or a set of semi-separable abilities (Elfenbein and MacCann 2017; Fiori and Antonakis 2011). Taxonomically, the nature of intelligence has been heavily informed by a factor-analytic tradition, in which new mental abilities are vetted based on how their covariation conform to various structural models. An early exemplar of this approach is Spearman’s (1904) principle of positive manifold which states all intelligence tests positively covary due to the operation of a broad mental capacity, often labelled as *g* or general cognitive ability.^1^ The idea of a singular intelligence was challenged by the search for fundamental, albeit overlapping, ability factors representing narrower content domains (e.g., verbal, numerical) or separable cognitive operations (e.g., perceptual organization, working memory) (Thurstone 1938), with the broadest distinctions made between fluid and crystallized forms of intelligence (Cattell 1943). This bottom-up approach views *g* as emerging from the interplay between fundamental abilities and implies the EI branches are distinct aptitudes that mutually reinforce one another over time (Joseph and Newman 2010; Van Der Maas et al. 2006).

Unitary and primary streams of intelligence research culminated in the landmark Cattell-Horn-Carroll (CHC) three-stratum theory of intelligence, which combines general and specific abilities into higher-order or nested-factor (i.e., bifactor) models of multifaceted capabilities existing at varying levels of abstraction (Beaujean 2015; Carroll 1993; McGrew 2009). The higher-order model posits a second-order factor or superordinate ability that directly participates in defining the nature of specific abilities by explaining their latent correlations. In contrast, the bifactor model suggests a general mental ability directly explains observed task performance independent of specific abilities. As the higher-order model is nested in the bifactor model, both models can be statistically transformed to the other leading many to believe they are equivalent (Reise 2012). However, a key distinction is whether specific abilities are conceptualized as mechanisms mediating the effects of g on subtest performance or as group factors explaining subtest performance independent of *g* (Beaujean 2015; Gignac 2008). Both higher-order and bifactor models treat intelligence as multifaceted and varying in breadth, but the bifactor model has the advantage of allowing intermediate and global abilities to exist at the same conceptual level—general ability and EI branches are neither “higher” or “lower” than the other but rather compete to explain task performance. Supporting this argument, the bifactor model fits intelligence data better than a higher-order structure in 90% of comparisons across multiple cognitive batteries and samples (Cucina and Byle 2017).

Similarly, there is evidence EI can be considered a broad intelligence alongside other general abilities (MacCann et al. 2014); yet, there is ambiguity as to whether and how branches should be combined or disaggregated. The three primary branches are only moderately intercorrelated (*ρ* = .34 to .55) (Joseph and Newman 2010) and moderately overlap with fluid and crystallized intelligence (*ρ* = .20 to .43) (Olderbak et al. 2019), which suggests EI branches and broader cognitive abilities are related but not fungible markers of a unitary ability. Multiple structural analyses support the poor fit of unidimensional models, but suggest several acceptable multidimensional or hierarchical arrangements. For example, Fan et al. (2010) conducted a factor analysis of the MSCEIT’s four branches on a pooled meta-analytic correlation matrix (*N* = 10,573; 12 EI ability subtests) and revealed the best-fitting model was a three-factor oblique model (RMSEA = .045; SRMR = .028, CFI = .97), rather than a unidimensional one (RMSEA = .096; SRMR = .082; CFI = .84). The best-fitting, 3-dimensional model had highly intercorrelated factors (*ϕ* range = .61 to .69), but not to a level to suggest emotional abilities fit a strictly unidimensional model. A comprehensive analysis evaluated MSCEIT’s placement within the CHC using five structural equation models and 21 manifest variables representing multiple general cognitive abilities (e.g., quantitative reasoning, fluid intelligence) (MacCann et al. 2014). A hierarchical model produced the best fit, with *g* as a second-order factor and EI as a first-order factor defined by perception, understanding, and management (RMSEA = .062; SRMR = .052; CFI = .977). This result places EI as a unitary ability operating at the same level as fluid intelligence and visual processing. However, both bifactor (RMSEA = .072; SRMR = .055; CFI = .973) and oblique eight-factor (RMSEA = .064; SRMR = .055; CFI = .976) models provide comparably good representation of the data, suggesting the EI branches could plausibly exist as semi-independent skills. Together, these illustrative examples suggest EI is not strictly unidimensional; rather, EI is better treated as a hierarchical set of abilities or as overlapping but not necessarily subordinate abilities.

**Figure 1:**
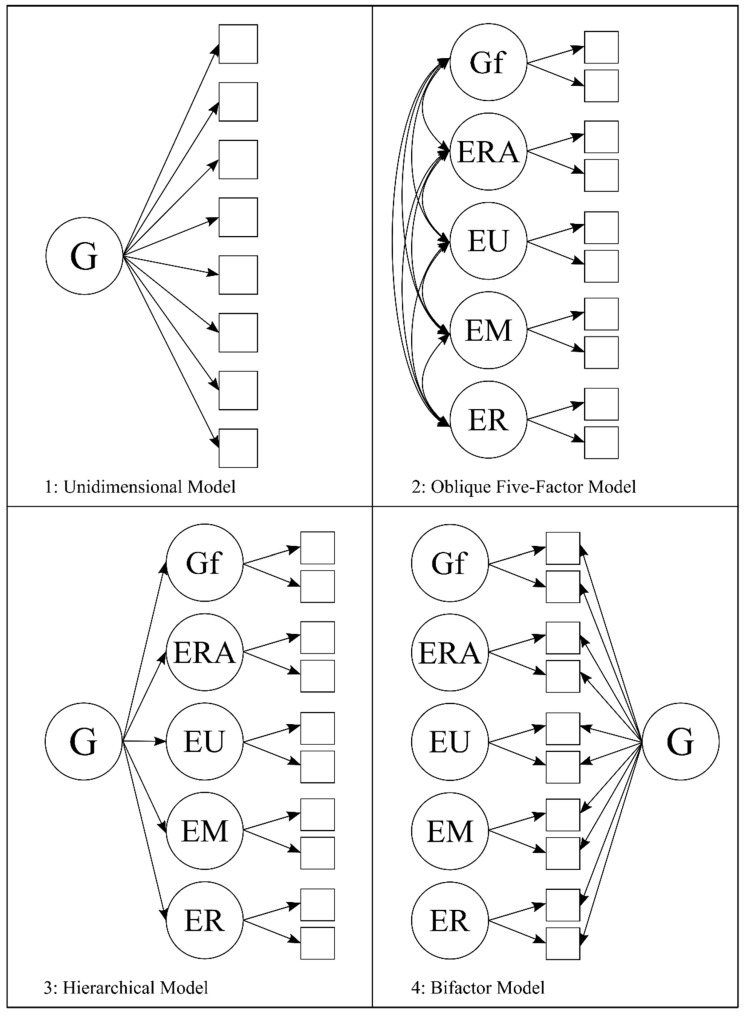
Simplified representation of tested models. G = General ability; Gf = fluid intelligence; ERA = emotion recognition ability; EU = emotion understanding; EM = emotion management; ER = emotion regulation.

Building on this work, we evaluate if the GECo fits the description of a broad, albeit multidimensional, intelligence in two ways. First, we evaluate the degree of positive manifold by testing if GECo’s branches converge with one another as well as fluid intelligence—a relatively pure marker of *g* (Floyd et al. 2003; Horn and Blankson 2005)—thus satisfying two of the three correlation criteria for considering EI an intelligence (Mayer et al. 1999). Second, we fit a variety of theoretical models consistent with the competing views of intelligence reviewed above. These structural models are illustrated in Figure 1 and include: (a) a unidimensional model in which all GECo and fluid intelligence (Gf) indicators load directly onto a unitary *g* factor (Model 1); (b) an oblique five-factor model that allows factors of Gf, emotion recognition, emotion understanding, emotion management, and emotion regulation to correlate freely (Model 2); (c) a hierarchical five-factor model in which the five factors from Model 2 load onto a second-order *g* factor (Model 3); and a bifactor model that has each indicator defining both a *g* factor and one of the 5 group factors described in Model 2 (Model 4). We note CHC theory affords intermediate positions for perceptual, verbal, and attentional abilities, which mirror skills in perceiving, understanding, and regulating emotional information (Schneider et al. 2016). Hence, all tested structural models imply GECo branches occupy their own position in the second stratum of CHC based on different cognitive operations.

#### Plausibility of Broad Subscales and General Factor Dominance

Structural evidence for multidimensionality suggests meaningful distinctions in specific abilities but does not indicate if such distinctions correspond to precise test scores. An unaddressed question in the structural modeling of EI batteries is whether the MSCEIT or GECo would be more appropriately scored and interpreted as global measures or broken down into specific subscales. Several practical applications arise for interpreting different EI branches, such as determining eligibility for specialized services, selecting individual into interpersonal jobs, designing problem-solving activities in educational settings, and formulating developmental goals and strategies.

The degree to which multidimensional solutions translate into appropriate decisions presumes EI batteries accurately quantify differences in broad abilities. While goodness-of-fit indices indicate a theoretical model reflects the data, they do not guarantee the solution produces strong, clear, and reliable factors. For example, when computing subscales from the bifactor perspective, EI branches will reflect variation on both an overall ability factor and more specific skills (e.g., emotional perception). The resulting effect is subscales may appear reliable but, in fact, the reliability is a function of the general rather than specific ability. This produces a counterintuitive finding where subscales exist but become so unreliable that the overall composite score is a better predictor of an individual’s true score on a subscale than the subscale score itself (Reise et al. 2013).

Beyond global fit indices, several complementary metrics assess the appropriateness, quality, and usefulness of hierarchical structures. These metrics judge the proximity of multidimensional to unidimensional solutions, factor salience, and reliability of subscale scores (Rodriguez et al. 2016b). First, two indices of appropriateness, explained common variance [ECV] and percentage of uncontaminated correlations [PUC], evaluate if a unidimensional model is “good enough” for the data despite structural evidence for multidimensionality. ECV indexes how much common variance is attributable to either a general or specific factor; whereas, PUC is the proportion of correlations among indicators attributed solely to the general factor. The PUC qualifies ECV, such that the biasing effects of a weakened general factor are less pronounced when PUC values rise. Presuming group factors justify multidimensionality, a related question of potency is whether factors univocally reflect the latent constructs they attempt to estimate. This is evaluated by factor determinacy (FD), the correlation between factor scores and the factor, and construct replicability (H), the correlation between a factor and an optimally weighted item composite. Both indicate the stability of a factor’s meaning in terms of producing accurate factor estimates and replicable latent variable specifications. Presuming the factors are *theoretically* well-defined for research, the next question is the precision of observed scores for scoring and interpreting an individual’s standing on general or specific skills. Several model-based reliability indices address if observed total scores reflect variation on a single latent variable (omega, omegaH) and, relatedly, whether subscale scores for each GECo branch (e.g., perception, understanding) reflect reliable variance both with and independent of the general factor (omegaS, omegaHS). Collectively, these indices allow practitioners and researchers to judge the usefulness of a multidimensional solution for subsequent psychometric analyses and applications. For instance, the indices indicate whether (a) variance in intellectual performance on EI assessments is due mostly to shared or distinct abilities, (b) SEM measurement models of EI branches will replicate in new contexts, and (c) overall EI scores and branch-specific scores yield reliable information. To our knowledge, no other investigations have thoroughly evaluated the measurement implications of treating an EI battery as a hierarchical construct containing both a general overarching ability and several intermediate subdomains presumed to arise from clusters of similar performance tasks.

### 1.3. Incremental Validity of EI Branches for Emotional Criteria

Both the structural model used to understand covariation patterns of tests (e.g., higher-order, bifactor) and the quality of scores derived from these tools (e.g., sum of unweighted and optimal weighting of subtests) have implications for interpretation. However, the practical value of distinguishing EI branches must be demonstrated by their utility in incrementally predicting criteria over one another and beyond individual differences in personality and intelligence (Fiori and Antonakis 2011). The question of whether EI branches are useful distinctions or subservient skills also relates to a “great debate” in the intelligence community about the practical role of narrow abilities (Kell and Lang 2017; Schneider and Newman 2015). The essence of the argument centers around whether specific abilities, like emotional understanding, remain valuable predictors after accounting for general ability and whether the usefulness of specific abilities depends on the specificity of the outcome (Beier et al. 2019).

The latter stipulation is key for EI because the incremental validity of specific skills may only be evident for specific criteria (Schneider and Newman 2015). Major reviews show EI is not a strong predictor of broad outcomes such as creativity, job performance, and leadership effectiveness (Harms and Credé 2010; Joseph and Newman 2010; Mayer et al. 2008). Rather, EI is a second-stratum ability. It is employed to solve emotional problems within a circumscribed space of affectively charged life experiences. These include coping with stress, sustaining motivation over time, establishing and maintaining close relationships, or adapting to rapidly changing situations with shifting social demands (Joseph and Newman 2010; Mayer et al. 2016). The aligned focus of predictors and criteria is consistent with the compatibility principle (Ajzen and Fishbein 1977), developed to account for inconsistent or weak relationships between attitudes and behavior. For instance, when researchers match specific cognitive abilities to criteria (i.e., perceptual abilities for jobs requiring quick, accurate processing of simple stimuli), they find support for incremental validity of specific abilities over g (Mount et al. 2008). Similarly, the emotion regulation branch of EI has a stronger association with job performance in emotionally laborious roles (e.g., customer service) which require one to remain calm, enthusiastic, and patient in dealing with others (Joseph and Newman 2010).

Within educational settings, theory suggests EI should be uniquely aligned with college adjustment and motivational criteria. When daily experiences of frustration, isolation, anxiety, enjoyment, and curiosity are attended to, understood, used, and effectively managed, the result is an enhancement of cognitive activities, personal happiness, and social functioning (Mayer and Salovey 1997). Drawing on this argument, in the present study we reason that students’ well-being and affective engagement are aligned with the information processing capacities of EI; whereas, accumulated academic performance in terms of grade point average (GPA) is aligned with general cognitive ability. Well-being refers to favorable evaluations of how life is going coupled with the frequency of positive compared to negative emotional states. Affective engagement captures emotional immersion in daily roles such as interest, excitement, and attachment to learning and school. Both share emotional overtones, but the former captures a holistic sense of happiness and the latter reflects energetic presence, investment, and connection to daily pursuits (Lam et al. 2014; Rich et al. 2010; Su et al. 2014). As emotions can be used as signals for navigating our environment, those high on EI will be better at detecting, understanding, and acting upon these signals to adjust daily efforts to improve quality of life.

A potential advantage of emphasizing specific EI abilities is understanding how affective signals inform adaptation: by reading and seeing emotions (perception), analyzing their significance (understanding), or acting upon them (regulation/management). It has been proposed emotion perception is needed to navigate interpersonal encounters by accurately decoding others’ intentions and thoughts (Schlegel 2020). Students who can ‘read others’ may be more socially responsive thus developing the deep connections needed for greater happiness (Diener and Seligman 2002). Once emotions are detected they must be accurately interpreted to usefully inform action. The understanding branch of EI is uniquely aligned with less biased decision making based upon accurate appraisals of the antecedents, responses, and consequences of emotional episodes (Alkozei et al. 2016; Hoerger et al. 2012; Mayer and Salovey 1997; Yip and Côté 2013). Thus, an emotionally knowledgeable student may be less affected by incidental moods and more accurate in forecasting which experiences will promote well-being. Finally, emotion regulation and management refer to processes used to redirect the spontaneous flow of negative and positive feelings in self and others (Gross 1999). Individuals high on EI are presumably able to fluidly alter emotions to create hedonic balance, avoid prolonged negative arousal, and attain mood-congruent aims such as performing a task or satisfying personal needs (Hughes and Evans 2018; Joseph and Newman 2010; Peña-Sarrionandia et al. 2015; Sánchez-Álvarez et al. 2016; Tamir 2016; Zeidner et al. 2012). For instance, a student facing a difficult course may subdue their worry by proactively framing everything as a challenge rather than hindrance, seeking help from friends, and accepting the fact they cannot know everything. By enacting multiple strategies to reduce rather than avoid negative states, a student high on regulatory branches is more likely to attain aims of enjoying school (hedonic needs) and mastering their profession (instrumental goals).

#### Using the S-1 Bifactor Model to Test the Predictive Validity of EI Branches

While many authors advocate for studying the unique properties of EI’s branches (Parke et al. 2015; Yip and Côté 2013), rarely has research accounted for the entanglement of specific EI abilities within a broader ability factor. As noted earlier, the bifactor model places general and specific abilities at the same conceptual level enabling the determination of their independent effects on outcomes. However, reviews of predictive bifactor models indicate numerous anomalous findings—abnormally large regression coefficients, wide standard errors, attenuated loadings, inadmissible solutions—which arise from using fixed rather than randomly selected and thereby interchangeable indicators (Eid et al. 2017, 2018). These issues can occur because the general factor lacks a strong theoretical anchor to stabilize its meaning. Consequentially, variability in indicator loadings leads most general factors to inadvertently be defined by different facets and items across studies (Eid et al. 2017, 2018).

**Figure 2:**
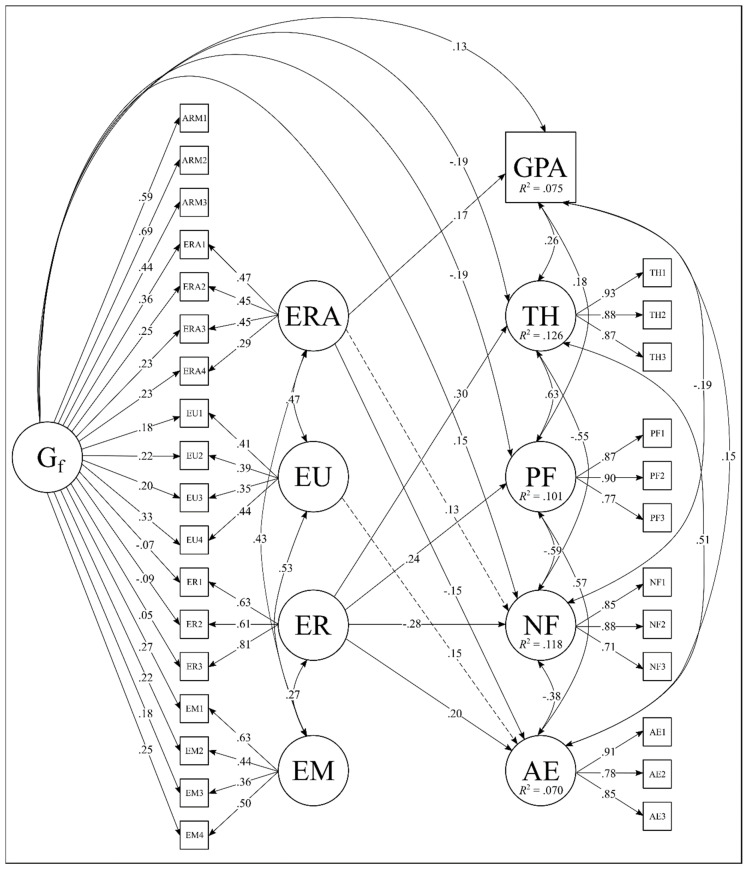
Bifactor (S-1) structural model of associations between latent factors representing general ability (fixed by fluid intelligence), GECo branches, well-being factors, affective engagement and manifest GPA. Parameter estimates are standardized beta coefficients. Statistically significant paths and correlations at *p* < .05 indicated by solid lines; marginally significant paths at *p* < .10 indicated by dashed lines. Gf = General ability-fluid intelligence referent; ARM*_i_* = Advanced Raven’s Matrices; ERA*_i_* = Emotion Recognition Ability; EU*_i_* = Emotion Understanding; ER*_i_* = Emotion Regulation; EM*_i_* = Emotion Management; TH*_i_* = Thriving; PF*_i_* = Positive Feeling; NF*_i_* = Negative Feeling; AE*_i_* = Affective Engagement. *i*: parcel indicator.

To prevent these issues, one group factor should be set as a reference indicator for general ability to which other group factors are compared (Eid et al. 2018; Heinrich et al. 2018). This technique is known as bifactor S-1 because there is one factor less than the factors considered. In the present study, fluid intelligence was chosen as the referent for general ability given empirical evidence for its near unity with *g* (Gustafsson 1984) and theoretical arguments that fluid intelligence captures earlier mental capabilities from which all other abilities accrue (Horn and Cattell 1966). All fluid intelligence performance tasks are used as indicators of the general reference factor and there is no specific factor for these indicators; all true score variance underlying fluid intelligence performance is reflected in the general ability factor. This results in an unambiguous a priori definition of the general factor (i.e., fluid intelligence tasks corrected for random measurement error), which does not change with addition of new performance tasks. The GECo branches are then contrasted statistically against fluid intelligence tasks and as regression residual factors arising from latent regression on the general reference factor. In this case, GECo branches reflect true score variance in specific EI skills unshared with general fluid intelligence factor (residual factors with a mean of zero per definition; see Eid et al. (2017) for full description). Beyond resolving interpretive ambiguities around representation of the general factor, setting a fixed referent removes linear dependencies which allow researchers to covary specific factors in testing unique predictive effects (Eid et al. 2018). A depiction of the S-1 model for the current study is presented on the left-hand side of Figure 2.

### 1.4. Study Aims

Building on the work of MacCann et al. (2014), we seek to conceptually replicate and extend a second-stratum understanding of EI by evaluating if specific GECo abilities provide unique information and reliable sub-scores in comparison to a general ability factor. First, in order to provide converging evidence that EI’s structure is transferable across instruments rather than localized to the MSCEIT, we examine if the GECo conforms to a simplified representation of the CHC theory with GECo branches treated as broad, second-order abilities parallel to fluid reasoning. Our aims are modest compared to MacCann et al. (2014), as we do not model a wide spectrum of broad cognitive abilities (e.g., short-term memory, visual processing) nor situate EI as a broad group factor within the second stratum of intelligence. Rather, we examine if GECo branches overlap with fluid intelligence and if the GECo is compatible with findings showing EI conforms to a hierarchical intelligence structure. Next, we extend past results by using fine-grained psychometric analyses to evaluate if shared variance across GECo branches and fluid reasoning can be reliably and accurately decomposed into overall and sub-branch scores. Finally, we apply a bifactor S-1 model to contrast the effects of specific GECo branches against general ability (as defined by fluid intelligence) in predicting academic performance, well-being, and affective engagement both in isolation and incrementally to personality. This model allows a rigorous test of the compatibility principle in which we expect distinct EI abilities to explain unique variance in emotional criteria (well-being, affective engagement) and general ability to explain unique variance in technical performance across courses.

## 2. Materials and Methods

Data were gathered as part of a department-wide assessment project between May 2016 and November 2018 from 1469 undergraduates attending a public university in the Northeast of the United States. We applied multiple carelessness response procedures to enhance data quality (DeSimone et al. 2015). First, we removed duplicate submissions (*n* = 125) and instances of missing data from one or more ability subtests (*n* = 340) some of which were overlapping cases. Second, participants indicating “slightly disagree” or “disagree” to the statement “In your honest opinion, should we use your data” were excluded (*n* = 87). Three, we included six instructed (e.g., “Click disagree to this item”), six bogus (“I am using a computer currently”), and six infrequency items (e.g., “I like to get speeding tickets”; Maniaci and Rogge 2014) and screened out anyone whose average fell above the mid-point for any of the three item sets (*n* = 108). Finally, we excluded participants completing the EI battery or remaining assessments in less than 15 min (*n* = 10; *M* = 46.37 min, *SD* = 22.2 min), resulting in a final analytic sample of 821 (~20% excluded for inattention).

Participants were predominantly female (82%) with an average age of 19.94 (*SD* = 3.13) and mixed racial composition (White = 47.4%; Hispanic = 24.1%, Black = 11.7%, Asian = 5.60%, Mixed = 6.34%). Participants represent all years of college standing, with most being freshmen (42.8%) and sophomores (24.7%), and encompass a variety of majors including psychology (*n* = 405), biology (*n* = 81), English (*n* = 32), athletics (*n* = 44), nursing (*n* = 22), and undeclared (*n* = 124).

### 2.1. Measures

#### 2.1.1. Fluid Intelligence

Participants completed the nine-item short-form of the Advanced Progressive Matrices Test (ARM) (Bilker et al. 2012), a non-verbal intelligence test consisting of a matrix of figural patterns with a missing piece. Students were presented a series of matrices with 8 response options that can be solved by deducing patterns and applying logical rules. Items were presented in the same order of increasing difficulty to all participants with a 6-min time limit. The ARM is less affected by culture and is known as a pure, but not perfect, indicator of general cognitive ability (Carroll 1993).

#### 2.1.2. Emotional Intelligence

The GECo is a 110-item measure providing four scores (Perceiving, Understanding, Regulating, Managing Emotions) and an overall EI score. The perception test is derived from the short form of the Geneva Emotion Recognition Test (GERT-S) (Schlegel and Scherer 2016) and contains 42 short audiovisual clips taken from a validated database of emotional expressions, the Geneva Multimodal Emotion Portrayals corpus (GEMEP) (Bänziger et al. 2012). Participants view 10 actors portraying 14 emotions (six positive, seven negative, and surprise) using the same pseudolinguistic sentence. Test-takers must correctly identify the expressed emotion from the 14 emotion words.

The understanding subtest contains 20 vignettes describing emotionally charged situations with correct answers derived from predicted emotional appraisal patterns according to the Component Process Model of emotion (Scherer et al. 2001). Participants need to correctly identify which emotion a person is likely to experience given a configuration of situational characteristics and cognitive evaluations surrounding an event. For example, the appraisal pattern for anger includes a situation with high novelty, a relevant but obstructed goal pursuit, and other-control. The 20 vignettes are comprised of two situations for anxiety, boredom, disgust, guilt, relief and shame and a single vignette for anger, irritation, contempt, fear, happiness, interest, pride, and sadness. Inclusion of subtly different pairings (e.g., anger, irritation) is done to purposely create greater task difficulty.

The emotion regulation subtest includes 28 vignettes describing situations of three broad categories of negative emotions: sadness/despair, fear/anxiety, and anger/irritation. Test-takers choose two of four options reflecting the actions or thoughts they would have in the situation. Responses contain two adaptive (i.e., acceptance, putting into perspective, positive refocusing, refocusing on planning, and positive reappraisal) and two maladaptive (i.e., blaming oneself, blaming others, rumination, and catastrophizing) cognitive regulation strategies (Garnefski et al. 2001). The adaptiveness of the strategy is rooted in empirical work showing which strategies are most likely to reduce the negative emotional states felt during the situation (Garnefski et al. 2001), with test-takers receiving one point for each adaptive strategy chosen.

Finally, the emotion management subtest consists of 20 interpersonal situations where the test-taker reads about another person experiencing an emotion from four broad categories: anger/irritation, fear/anxiety, sadness/despair, and inappropriate happiness (e.g., schadenfreude). Test-takers are asked to choose one of five behavioral strategies (competing, collaborating, compromise, avoidance, and accommodation) which would best handle a conflict and appease the other person given a set of situational parameters, such as power differences, norms, stakes, incentives, and competing interests.

#### 2.1.3. Big Five Personality Traits 

The Mini-International Personality Item Pool–Five-Factor Model (Mini-IPIP) (Donnellan et al. 2006) is a 20-item measures assessing the five personality dimensions of extraversion, openness, conscientiousness, neuroticism, and agreeableness, with imagination used in place of openness to experience. The scale consists of a series of four statements per each Big Five trait that participations rated on a 7-point scale ranging from 1 (*Very Inaccurate*) to 7 (*Very Accurate*).

#### 2.1.4. Subjective Well-Being

Well-being is most commonly assessed using Diener’s (1984) tripartite formulation of positive affect (PA), negative affect (NA), and life satisfaction, which captures the frequency of positive and negative affective experiences along with global evaluation of one’s overall life (Diener et al. 1999). Here, we measured life satisfaction with the expanded Brief Inventory of Thriving (BIT) (Su et al. 2014) and PA/NA using the Scale of Positive and Negative Experience (SPANE) (Diener et al. 2010). The BIT is a 10-item measure capturing a broad range of well-being constructs including subjective well-being, supportive relationships, interests, meaning, master, autonomy, and optimism (e.g., “My life is going well”, “My life has a clear sense of purpose”). Ratings are provided for life in general using a seven-point scale (1 = *strongly disagree* to 7 = *strongly agree*) and are summed into a single barometer of “how life is going” (Su et al. 2014). The SPANE is a 12-item measure of the frequency rather than intensity of positive (six items; e.g., joyful, happy, contented) and negative (six items; e.g., sad, angry, afraid) affective experiences. Ratings are provided based on the past four weeks using a seven-point scale (1 = *very rarely or never* to 7 = *very often or always*) and are averaged to compute separate positive feelings (PF) and negative feeling (NF) scores.

#### 2.1.5. Affective Engagement

The affective engagement sub-scale from the Student Engagement Scale (Lam et al. 2014) is a 9-item measure of the degree to which students are interested and emotionally attached to learning and their institution (e.g., “I enjoy learning new things in class”, “I think learning is boring”). The scale was validated across 3420 students from 12 countries and shown to be internally and temporally consistent while also converging with other engagement scales and correlating with support, instructional practices, emotions, and academic performance. Ratings are provided on a seven-point frequency scale (1 = *never* to 7 = *every day*).

#### 2.1.6. Cumulative Grade Point Average

Overall, academic achievement was operationalized as student’s cumulative grade point average attained from self-reports and verified with consent from electronic transcripts during the final semester of college. The average time elapsed between the initial assessment battery and final cumulative grade was 23 months (*SD* = 13.7).

### 2.2. Analyses

#### 2.2.1. Model Estimation and Comparison

All analyses were conducted in R version 3.6.2 (R Core Team 2019) using *semTools* (Jorgensen et al. 2020) and *lavaan* (Rosseel 2012). Models were estimated using the robust maximum likelihood estimator (MLM) which provides standard errors and model fit tests robust to non-normality. No missing responses were present given data pre-screening. In the first stage of analyses, we fit all four specified models (see Figure 1). To keep the ratio of observable measures to latent constructs tractable while also stabilizing the factor solutions, we modeled each GECo branch using facet-representative parceling in which items sharing secondary face-relevant content are bundled into parcels based on conceptual overlap (Bagozzi and Edwards 1998; Little et al. 2002).^2^

Parceling was done by grouping item content based on shared emotional families (e.g., fear, anger) or approximate location within the affective circumplex when a GECo branch included problem solving items spanning more than 4 discrete emotions (Yik et al. 2011). This resulted in the 14 emotions for ERA being grouped into four parcels for recognizing high activation, negative valence emotions (e.g., anger, disgust; *n* = 18); higher activation, positive valence emotions (e.g., pride, joy; *n* = 9); lower activation, positive valence emotions (e.g., relief, interest; *n* = 9); and lower activation, negative valence emotions (e.g., despair, sadness; *n* = 6). Similarly, the 14 emotions for EU were grouped into four parcels for understanding appraisal profiles of highly active, negatively valence emotions targeted at others (e.g., anger, disgust; *n* =5); highly active, negatively valence emotions targeted at circumstances and self (e.g., anxiety; guilt; *n* = 7); low activation, negatively valence emotions (e.g., boredom, sadness; *n* = 3); and positively valence emotions (e.g., happiness, pride, interest; *n* = 5). The four emotional families for emotion regulation (i.e., anger/irritation, fear/anxiety, inappropriate happiness, sadness/despair) allowed the even division of item sets (*n* = 5) into four parcels. Finally, we retained the three emotional families for emotion regulation (i.e., anger/irritation, fear/anxiety, sadness/despair) to form three parcels (*n* = 8–10). For fluid intelligence, we used a triplet split where every third item was assigned to the same parcel (e.g., first parcel contains items 1, 4, and 7). This ensured parcels were balanced (one parcel would not contain “later” items for which a time limit may have restricted performance) and item sets would be of approximate equal difficulty. We followed a similar serial parceling strategy for well-being and motivational outcomes to produce three parcels per factor whereas the abbreviated Big Five scales (4 items per factor) were modeled using item responses. The correlation matrix of parcels used in all subsequent SEM models is available as a supplementary table (Table S1).

All structural models are partially nested and thus most can be compared using chi-square difference (Δχ^2^) tests to guide model choice (Brunner et al. 2012). However, because the chi-square (χ^2^) test of exact fit and, by extension, nested fit tends to be sensitive to sample size and minor misspecifications, we also relied on the following common goodness-of-fit indices to compare global fit: the comparative fit index (CFI), the Tucker-Lewis Index (TLI), the root mean square error of approximation (RMSEA), and the standardized root mean square residuals (SRMR). According to typical interpretation guidelines (Hu and Bentler 1999; Marsh et al. 2005), values greater than .90 and .95 for the CFI and TLI, respectively, are considered to indicate adequate to excellent fit to the data; whereas, values smaller than .08 or .06 for the RMSEA and SRMR, respectively, show acceptable and good fit (Brown 2015). We also considered the Akaike (AIC) and Bayesian (BIC) information criteria as metrics with lower numbers suggesting better relative fit given a tradeoff between quality and parsimony.

Given more complex models often produce better fit, several authors recommend complementary comparisons of parameter estimates, bifactor indices, and theoretical considerations to dictate choice among equally optimal models (Brown 2015; West et al. 2012). In comparing first-order to bifactor models, it is important to note bifactor (group and general) estimates are often weaker than first-order factor models, which is due to the disaggregation of indicator-level covariance into estimates of two separate factor sets (general and group) rather than a single one (Reise 2012). Specifically, in first-order models, all the shared covariance among a specific subset of indicators is absorbed into the first-order factors. In contrast, in bifactors models, all shared indicator variance (including specific subsets under consideration) is absorbed into the general factor leaving “left over” covariance to be absorbed into group factors. As such, the critical component of this comparison is the observation of a sensibly defined general factor accompanied by at least some well-defined group factors.

#### 2.2.2. Psychometric Evaluations

The *BifactorIndicesCalculator* (Dueber 2020) was used to calculate several additional indices of model usefulness discussed in the introduction (see Rodriguez et al. 2016b for more details). ECV and PUC explain how much common variance is due to different factors and what percentage of indicator correlations reflect only the general factor. We report two ECV indexes for general and group factors (Stucky and Edelen 2014). The within-domain ECV (ECV_GS) is the proportion of common variance in indicators due to the general factor. ECV_GS signifies how much variance within group factors is driven by general ability. The specific-dimensions ECV (ECV_SG) computes the strength of a specific factor relative to all explained variance across indicators, even those not loading on the specific factor of interest (Stucky and Edelen 2014). Consequentially, ECV_SG values sum to 1 and dissect how much common variance is due to the general factor versus group factors. Note ECV indices are identical for the general factor but differ for group factors.

FD ranges from 0 to 1, with values above .80 providing factor determinacy for research (Gorsuch 1983) and .90 as truthful substitutes (Grice 2001). In a related vein, H also ranges from 0 to 1 and is the population squared multiple correlation from regression the construct on its indicators with high values suggesting a well-defined construct likely to replicate in future studies. *H* values should minimally exceed .70 (Hancock and Mueller 2000) but ideally be above .80 (Rodriguez et al. 2016a). Together, FD and H show how well the indicators approximate their latent variables. Omega (*ω*) indicates the proportion of construct score variance (of the general and group factors) relative to the total amount of variance and is the latent variable analog to coefficient alpha. Omega hierarchal (*ω*_H_) represents variance in the model attributable to the general factor in the bifactor model (independent of group factors), whereas omega subscale (*ω*_S_) estimate represents the proportion of reliable variance of the general factor and a group factor. Omega hierarchical subscale (*ω*_HS_) is just the proportion of reliable variance of a group factor after removing variability due to the common factor (Reise et al. 2013). These values give a sense of overall and unique variance attributable to factors and are interpreted as the reliability of total and subscales scores.

Most effect size recommendations for bifactor indices are based on first-order factor models rather than relative comparisons to past bifactor findings. For instance, suggested values for FD and H assume intact factors which may provide an especially high ceiling for group in bifactor models which represent residualized and thus shrunken constructs. As an alternative point of comparison, we compare current results to the bifactor indices associated with 50 past multi-factor solutions from 50 previously published correlation matrices (Rodriguez et al. 2016a). Adopting a similar strategy to Gignac and Kretzschmar (2017), we use the *rsnorm* function in R to simulate effect size distributions with 10,000 observations using Rodriguez et al.’s (2016a) descriptive and skewness results (presented in their Table 2) and the *quantile* function to extract the 33rd and 66th percentile for all indexes. Alternative benchmarks are presented in Table 1 with values demarcating what might be considered small (<33rd percentile), medium (33rd < index < 66th percentile), and large (>66th percentile) effects. These effects are relative comparisons for what is typical given the state of current psychological assessment.

## 3. Results

Descriptive statistics and correlations for all measures are shown in Table 2. Pearson correlations among all GECo scales suggest a positive manifold (all positive, non-trivial correlations) except for emotion regulation (average *r* = .20). Emotion perception, understanding and management had significant associations with fluid intelligence (average *r* = .29) whereas regulation did not (*r* = −.03), a finding consistent with past research on the GECo (Schlegel and Mortillaro 2019) but not the MSCEIT (MacCann et al. 2014). The overall GECo score overlaps with fluid intelligence (*r* = .35) which, based on a Steiger test of dependent correlations, is larger than the overlap with openness (*r* = .17; *t* = 4.13, *p* < .001) and agreeableness (*r* = .17; *t* = 3.86, *p* < .001). As a supplementary analysis, we ran a more stringent test of discriminant validity by regressing error-free latent versions of each GECo branch on latent representations of fluid intelligence and the Big Five (full results in Supplemental Table S2). Fluid intelligence and personality predict moderate amounts of variance in GECo scales (multiple *R* range = .45 to .61) with the largest effects for fluid intelligence on emotion management, understanding, and recognition (*β* range = .41 to .52) and neuroticism on emotion regulation (*β* = −.36). These effects are smaller than parallel findings for the MSCEIT (Fiori and Antonakis 2011) which suggests some overlap but satisfactory distinction to show operation of unique skills. Fluid intelligence and the more cognitively loaded GECo branches (perception, understanding, management) all correlate with GPA whereas regulation is significantly related to well-being and affective engagement.

### 3.1. Structural Models

Table 3 presents indices for the four alternative models and one modified bifactor solution. Model 1 (one-factor CFA) fit the data very poorly and was considerably worse compared to the other three models, with TLI and CFI being much lower than .90 and the highest AIC and BIC values. Model 2 (five-factor oblique CFA model) yields a clearly superior fit (χ^2^ = 117.99, *p* = .659, CFI = 1.00, RMSEA = .00). Standardized factor loadings for parcels range from *λ* = .37 to *λ* = .83 (*Mdn λ* = .53), showing each factor is adequately but loosely defined. Significant correlations between first-order factors range from *r* = .24 (ER and EM) to *r* = .63 (EU to EM; *Mdn r* = .55), with all EI branches except regulation correlating with fluid intelligence (*r* range = .42 to .55). Model 3 (hierarchical) also provides excellent fit (χ^2^ = 146.34, *p* = .16) with a moderately well-defined second-order factor (*Mean λ* = .613); however, a scaled chi-square different test (Satorra and Bentler 2001) indicates Model 3 is less preferred to Model 2 (${\Delta \chi }_{\left(5\right)}^{2}$ = 30.33, *p* < 0001). Close inspection of residual correlations between the model-implied and actual oblique factor associations indicate the hierarchical model underpredicts the association between emotion regulation and management (Δ*r* = −.17), but all remaining correlation residuals between models are negligible (e.g., −.10 < Δ*r* < .10) (McDonald 2010). Thus, while a superordinate ability does not capture all the exact associations between GECo branches and fluid intelligence, the small residuals in combination with good fit supports EI as a hierarchically structured with abilities operating at various levels of generality. Finally, the bifactor model also provides excellent fit (χ^2^ = 117.99, *p* = .278) and is marginally better than Model 4 (${\Delta \chi }_{\left(13\right)}^{2}$ = 20.81, *p* = .08) but, based on the AIC and BIC, is less preferable to Model 2. Again, results suggest the presence of a general factor but fall slightly short of the oblique solution due to a single residual correlation. To test this hypothesis, we fit a modified bifactor model allowing emotion regulation and management to covary (see Model 5 in Table 3). This provided a significant gain over Model 4 (${\Delta \chi }_{\left(1\right)}^{2}$ = 26.44, *p* < .001) and fit very well (χ^2^ = 100.66, *p* = .84, CFI = 1.00, RMSEA = 1.00). While information indices favor Model 2 due to simplicity (fewer *df*), we opted to move forward with Model 4 given broader empirical support for *g* as a global construct contributing to all tasks requiring cognitive processing (Carroll 1993) and because bifactor indices (see below) along with residual and parameter analyses support a common factor running through a majority of indicators. Further, from a usability standpoint, the bifactor serves as a suitable compromise between theories emphasizing general versus specialized abilities by allowing both categories to be modeled in subsequent analyses.

### 3.2. Psychometric Analyses of a Bifactor Model

Table 4 shows the loadings of parcels on both the general factor and the five broad abilities for the bifactor solution. Except for ER1 and ER2 parcels on the general factor, all loadings are significant. The general factor is defined by ERA (*λ* = .32–.47; *M* = .39) and EM (*λ* =.30–.50; *M* = .39) followed by EU (*λ* = .33–.50; *M* = .38) and GF (*λ* = .26–.47; *M* = .36) with virtually no contribution from ER (*λ* = −.01–.12; *M* = .04). Further, all group factors have moderately sized loadings on specific abilities (GF *M_λ_* = .45; ERA *M_λ_* = .31; EU *M_λ_* = .27; EM *M_λ_* = .37) indicating the specific GECo branches have an incremental impact on their corresponding parcel scores over and above general ability; emotion regulation, in particular, has uniformly large loadings suggesting a tightly defined domain (ER *M_λ_* = .68). Together, this suggests shared variance in performance tasks is split across general ability and group-based factors, general ability is equally defined by fluid intelligence and ER branches suggesting a type of general reasoning, and emotion regulation is not related to ability in the same way as other GECo branches.

Bifactor indices for the overall ability and specific factors are also presented in Table 4. The model has a relatively large PUC (.84) and small average relative bias of 1% (Rodriguez et al. 2016b), suggesting a simplified unidimensional model would not bias parameter estimates for general ability. However, the general factor’s ECV is smaller compared to other bifactor models (.40; see Table 1) with a majority of the common variance (60%) spread across the independent effects of ER (.25), GF (.11), EM (.10), ERA (.07), and EU (.06). ECV_GS values show general ability explains no variability in emotion regulation but upwards of 64% of differences for emotion understanding. This also implies at least 33% of the performance differences for each EI subtest is attributable to specific abilities. Together, results suggest modeling EI and fluid intelligence together as a unitary ability would not bias the definition of the general factor but a non-trivial amount of unique variance in each EI branch would be lost in the process.

However, the degree to which GECo’s multidimensionality can be fully captured is suspect. FD values for all specific abilities are below .90 with only the general factor and emotion regulation being above .80. Further, H values range from .27 for emotion recognition to borderline values for general ability (.72) and emotion regulation (.77). The average H value for group factors (*M* = .44) suggests low replicability of latent specification of GECo branches, a typical finding for narrow domains in most bifactor models (see Table 1). Results suggest overall ability and emotion regulation are moderately well-defined and estimable, but caution is needed in modeling emotion recognition, understanding, and management.

The omega coefficient for the total score (*ω*) is .78 and the omega hierarchical coefficient (*ω*_H_) is .59 suggesting about 2/3rds of the reliable total score is attributable to the general factor. Omegas for subscale scores range from .53 (understanding) to .73 (regulation) suggesting subpar to adequate reliability for broad abilities. However, after partitioning variance explained by the general factor, the omega hierarchical subscale coefficients (*ω*_HS_) show less unique reliable variance for GECo branches ranging from 18% (understanding) to 77% (regulation). *ω*_HS_ can be considered an index of unique latent variable strength (Gignac and Kretzschmar 2017) with Table 2 suggesting emotion regulation is a “strongly” defined skill while emotion recognition and emotion management have “medium” effects and can be considered unique EI dimensions even if values are too low to allow rendering of reliable composite scores. Overall, 76% of reliable variance across indictors was attributable to the general ability factor (.59/.78), 19% to group factors (.78–.59), and 5% is due to error suggesting most reliable differences in unit-weighted composite scores are due to overall ability. Based on these results, reliable scores can only be produced for overall ability and an emotion regulation score.

### 3.3. Bifactor S-1 Predictive Models

The multivariate bifactor S-1 regression model with all outcomes regressed on fluid intelligence as a referent for general ability and 4 overlapping GECo abilities, as visualized in Figure 2. As ARM items load exclusively on the general factor it retains the same meaning as a fluid intelligence factor modeled alongside separate EI branches in a correlated first-order factor (see Eid et al. 2017 for more formal presentation). Only significant paths and correlations are displayed. The bifactor structural model fit well, χ^2^ (731) = 1764.26, *p* < .001; RMSEA = .042; CFI = .929; TLI = .917. Latent correlations among GECo branches range from .27 to .53 which shows several EI branches still share residual true score variance after partialling out fluid intelligence (range from 8% to 25%). The loading pattern for EI branches also show greater specificity and better definition compared to the classic bifactor model (H range .43 to .76, *M* = .57; FD range .70 to .88, *M* = .77) as the general ability shifted to be defined by fluid intelligence. Both a fluid intelligence general ability (*β* = .131) and, unexpectedly, emotion recognition (*β* = .167) accounted for significant variance in GPA thus supporting the expected role of mental ability in academic performance. While not predicted, fluid intelligence general ability is associated with less thriving, positive feelings, and more negative feelings suggesting a smarter but sadder effect. For the unique effects of GECo abilities, emotion regulation is related to all well-being indicators and greater affective engagement while emotion understanding is marginally (*p* = .08) associated with greater affective engagement and emotion recognition is marginally related to higher negative feelings (*p* = .06) and lower affective engagement. This result indicates certain EI branches contribute incrementally to adjustment above fluid general ability, but not always in the expected direction with more contributions arising for emotional engagement as compared to emotional well-being. A reviewer suggested possible suppression effects between EI subscales in predicting GPA. To probe further we ran a series of supplementary commonality analyses which suggest suppression effects are negligible (see Table S3).

Table 5 presents standardized beta weights for the S-1 bifactor model which includes the Big Five as latent predictors orthogonal to fluid general ability but covaried with EI branches and one another. The model provides acceptable fit, χ^2^ (1114) = 2057.62, *p* < .001, CFI = .926, TLI = .915, RMSEA = .033. Controlling for the Big Five, both fluid general ability and emotion recognition remain significant predictors of GPA while emotion regulation becomes a significant negative predictor. For well-being indicators, emotion regulation fell to non-significance for positive and negative feelings and remained marginally significant for thriving (*p* = .06), suggesting personality plays a more prominent role in eudemonic and emotional well-being compared to emotion regulation. Finally, emotion regulation and recognition remained significant but opposing predictors of affective engagement. Taken together, findings partially support the unique role of specific GECo skills in predicting affective and well-being criteria with stronger evidence for affective engagement.

## 4. Discussion

Overall, the results of the current study suggest the perceptual, understanding, and emotion management subscales of the GECo can be represented by a nested hierarchical (i.e., bifactor) structure with a general ability factor driving cognitive performance and unique factors accounting for specific emotional skills. Bifactor indices and loadings further support this split with much of the reliable variance in understanding, recognizing, and managing others’ emotions being absorbed into a general ability factor. In contrast, the regulating emotions skill fell outside this bifactor structure and does not meet a hierarchical definition of intelligence but has enough reliability to be measured as a standalone subcomponent. Evident by predictive bifactor models, emotion regulation offers the greatest incremental validity in predicting well-being and motivation above general fluid ability and the Big Five personality traits.

### 4.1. Structural Evidence of the GECo in Relation to Fluid Intelligence and the MSCEIT

A majority of the GECo branches overlap as strongly with one another as they do with fluid intelligence, satisfying two correlational criteria for considering EI as a type of intelligence (Mayer et al. 1999). Oblique and bifactor models fit the data equally well with information indices favoring an oblique structure. The GECo assessment captures the branches of an intertwining intelligence—individuals skilled at detecting patterns in abstract figures can more easily understand different emotions and, given this knowledge, endorse effective conflict management tactics. To what degree these associations are best explained by broader or narrower capabilities is less discernible from factor analytic evidence alone (van Bork et al. 2017). In such situations, theory and alternative evidentiary sources should be considered along with fit indices (Murray and Johnson 2013). From these data, a bifactor model, consistent with Carroll’s thinking on intelligence (Beaujean 2015), seems preferable. The indices show that a general factor explained 66% of the variance across subscales and captured a majority of reliable variance in scale scores. This representation is advantageous for modeling both general and primary abilities at the same level of abstraction rather than presume one set takes precedence, and such a model requires only limited changes as predicted by CHC theory (i.e., addition of other second-stratum factors) (McGrew 2009).

For convergent validity, the pattern of overlap with fluid reasoning is quite uniform across most GECo branches whereas the MSCEIT’s overlap with cognitive ability is more strongly driven by emotion understanding (Joseph and Newman 2010). For divergent validity, the overlap between GECo branches, cognitive ability, and personality does not suggest redundancy. Past research shows disattenuated multiple *R*’s between individual differences and MSCEIT branches ranging from .49 to .81, with some of the largest overlap occurring for agreeableness, openness, and conscientiousness (Fiori and Antonakis 2011; Schulte et al. 2004). In contrast, GECo branches all have corrected multiple *R*s less than .60, with effects predominantly driven by fluid intelligence. This result suggests the GECo branches are somewhat saturated by cognitive ability (as expected) but can still explain unique variance in outcomes. Finally, in terms of criterion validity, the GECo’s total score does not directly overlap with well-being which contrasts with the MSCEIT’s small associations with life satisfaction (Mayer et al. 2008). This result is qualified by the emotion regulation branch being moderately correlated with well-being, but structural evidence suggests regulation should not be included into an overall EI score. Together, findings suggest the GECo may provide a viable and freely available alternative to the MSCEIT in terms of nomological networks, but more research is needed to understand diverging criterion coefficients.

### 4.2. Emotion Regulation: Distinct Skill, Trait EI, or Methodological Artifact?

Emotion regulation emerged as an importance predictive branch of EI but did not fit in a hierarchical intelligence structure. It is plausible emotion regulation falls into a distinct territory akin to intrapersonal intelligence (Gardner 1983), or unique skills of discriminating internal feelings, attending to their sources, and using this information to guide action. This division is consistent with early theorizing on the nature of EI (Salovey and Mayer 1990), and multiple self-report EI scales (Niven et al. 2011; Pekaar et al. 2018). As implied by a recent review (Elfenbein and MacCann 2017), the positive manifold between EI sub-domains may be more reflective of basic capacities in reading and understanding other people’s emotions as opposed to our own.

There is theoretical and biological precedent for subdividing EI ability into dual tracks of intellectual reasoning about emotional information in self and others. First, a number of contemporary intelligence theories are not hierarchical and focus on multiple independent abilities or processes, such as Sternberg’s triarchic theory (Sternberg 1984), Gardner’s theory of multiple intelligences (Gardner 1983), and the planning, attention, and simultaneous-successive processes theory (Naglieri and Otero 2012). As emotion regulation and management partially but weakly overlap (*r* = .15), it is possible EI fits a decoupled version of the process overlap account of intelligence which holds that g (and other abilities) is the result of domain-general executive processes (Kovacs and Conway 2016). A general EI factor may be viewed as a composition of several distinct executive processes instead of a unitary ability. There also is neurological evidence for self-knowledge being processed through different neural mechanisms and brain regions compared to social knowledge (David et al. 2006; Vogeley et al. 2001). Awareness of dynamic relationships between the self, one’s surroundings, and other agents is an on-going task of the perceptual system and seems essential for separating personal actions from outside behaviors and events (Critchley et al. 2004; Damasio 2010). Finally, evolutionary psychology suggests we have distinct cognitive capacities for self-awareness and social perception, which collectively help us learn how to act and feel around others while also making wise choices about who to befriend and avoid (Buss 2008; Dunbar 2009; Gallup 1998).

A second possibility is emotional regulation is more akin to a competency or trait-oriented conceptualization of EI which mixes intelligence and personality (Hughes and Evans 2018; Soto et al. 2020; van der Linden et al. 2017). Unlike neuroticism which reflects enduring tendencies to feel bad or stressed across situations, a capacity or trait EI model suggest the capability or efficacy to regulate emotions and moods when the situation calls for it. For instance, someone may generally feel anxious (high neuroticism) or lack ability to identify and differentiate emotional states (low on some EI abilities) but can still calm themselves if needed to complete a specific task (e.g., presentation, resolve a conflict). As a competency, emotional regulation may have moderate relations with ability to process information and tendency to feel a certain way yet hold enough unique information to capture a functional proficiency to alter emotional states to attain hedonic, instrumental, or relational goals (Hughes and Evans 2018; Soto et al. 2020). This is suggested by our data on the improved fit for the modified bifactor model where regulation still covaried with emotional management even after accounting for general mental ability. This overlap may suggest a type of “practical” or “procedural” knowledge in managing emotions which uniquely joins these two branches. Future research might draw upon integrative EI models (e.g., Vesely Maillefer et al. 2018) to empirically test the degree to which the GECo regulation scale is jointly determined by trait, process, and ability-oriented conceptualizations of EI. Finally, there may be methodological reasons for the regulatory branch’s divergence. The questions within this section primed participants to answer based upon knowledge of what is correct versus preferred (Freudenthaler et al. 2008). The current data show regulation overlaps most strongly with neuroticism (*r* = −.30), suggesting those predisposed to experience negative affect are more likely to endorse maladaptive regulatory strategies across vignettes. Regulation also has small associations with conscientiousness (*r* = .17), extraversion (*r* = .15), and openness (*r* = .11) suggesting personality relates to responses. However, emotion regulation also shows the most incremental validity beyond personality and, comparatively speaking, has the same degree of overlap with emotional stability as MSCEIT’s regulatory branch with agreeableness (*ρ* = .30) (Joseph and Newman 2010). Future research on the GECo would benefit from experimental manipulations of response instructions to parse whether response variation is driven by abilities or tendencies. If the regulatory branch became more saturated by cognitive ability and less by neuroticism when items are framed as providing a best response (e.g., what “should” be done), divergence in GECo branches may largely be methodological rather than meaningful.

### 4.3. Predictive Effects and Alignment with Emotional Engagement

Current results reinforce meta-analytical findings on the role of emotional regulation as the “engine” driving key outcomes above cognitive ability and personality (Joseph and Newman 2010). Regulation was the only EI branch to predict all well-being and motivational criteria after controlling for general ability and other EI branches. While this outcome is partially due to its independence from intellectual reasoning, we note the bivariate effects for regulation and multiple outcomes are in the medium to large range (Gignac and Kretzschmar 2017) speaking to its direct importance in leading a happy life. Furthermore, even after controlling for the Big Five personality traits, emotion regulation is marginally connected to thriving and predicts emotional absorption in school.

Beyond regulation, residualized factors of emotion understanding and recognition contribute to affective engagement, but in opposing directions and with weaker effects. High EI may promote emotional reactions linked to greater task investment, such as interest and excitement, via effectively choosing experiences and events that will make ultimately make us happy (understanding) and finding joy in the mundane (regulation). However, perception seems to diminish affective engagement, possibly as a result of the “curse of hypersensitivity” (Schlegel 2020), whereby accessibility to emotional information can compromise health and social effectiveness when it is threatening, abundant, or conflicting (Schlegel 2020). Our findings feed recent calls to understand the contextual moderators of perception (Schlegel 2020), by suggesting too much skill in reading emotions may be detrimental to one’s interest and emotional connection to school.^3^

Finally, we note the emotion management scale appeared to be of little predictive use in this study, potentially due to a lack of inclusion of interpersonal outcomes focused on building relationships, working through conflict, or navigating social networks. High EI individuals are rated by others as more socially competent and collaborative (Brackett et al. 2006) with the emotion management branch of the MSCEIT linked to a sensitive and helpful reputation (Lopes et al. 2005). We expect interpersonal consequences of EI to be uniquely driven by skills in managing other’s emotional states and should be included in future GECo research.

In terms of GPA, results corroborate general ability as the driver of academic performance. While emotion understanding, management, and recognition all significantly correlate with GPA, these effects were reduced or fell to non-significance when controlling for a global fluid reasoning factor. The one exception was emotion perception which remained significant even after accounting for the Big Five personality traits. Interestingly, these results contradict a recent meta-analysis which found perception had the *weakest* association to GPA and concluded, “…the lower two branches of ability EI (emotion perception and facilitation) provide little to no explanatory power for academic performance over intelligence and personality” (MacCann et al. 2020, p. 19). Current results suggest a very different conclusion: perception has the strongest association with GPA beyond intelligence and personality. Given most studies included in the meta-analysis (MacCann et al. 2020) used the MSCEIT, which has been shown to have inconsistent correlations with other measures of emotion recognition (Roberts et al. 2006), it is not surprising that findings do not generalize to the GECo’s recognition tasks which uses dynamic, postural, and multi-sensory items with a forced choice format rather than emotion ratings on a Likert scale. Another source for the discrepancy could be that past research often used raw EI scores to represent broad and specific abilities which are unrefined estimates of targeted constructs. Our results suggest only a third of the variance in EI branches could be attributed to specific emotional skills above general ability and, furthermore, these effects are not reliably captured in raw scores. We would not expect models in which EI branches are included as disaggregated latent predictors to produce results which closely match studies using multiple regression with observed scores.

It is unclear why emotional perception aids GPA. One possibility is that emotional perception is related to more general cognitive operations involved in quick and efficient information processing such as better sensory discrimination (Schlegel et al. 2017). The same kind of acuity for detecting subtle changes in faces and voices may also be involved in deducing patterns in numbers and reading written passages. Second, emotion perception is associated with accurate deduction about one’s current standing and understanding of course material. Students may perform better in courses with presentations, oral exams, and team products by better adapting to social signals of poor performance, such as a confused friend or disappointed professor. The strength of negative signals may spur emotionally perceptive students to greater action when they fall behind. Finally, people who are emotionally perceptive may also acquire more support because they are easier to work with and more likely to respond to feedback.

Finally, results show the greatest incremental power for different EI branches came down to emotional immersion in specific tasks as opposed to how people feel about life in general. EI proponents link emotional competence to a number of life outcomes (Nathanson et al. 2016), yet reviews and studies show the MSCEIT’s effects on adaptive forms of coping, well-being, and social relations are somewhat inconsistent, small in magnitude, and attenuated when controlling for personality and cognitive ability (Bastian et al. 2005; Rossen and Kranzler 2009; Zeidner et al. 2012). Based upon the compatibility principle of aligning predictors and criteria, our results suggest inconsistencies of EI’s importance may partially arise from a focus on criteria which are multiply determined and broad in scope. Rather, EI branches show more predictive value when narrowing the focus from feelings about everything in life to emotional investments in specific roles. Emotionally competent individuals may experience life dissatisfaction but can still regulate emotional states when needed for short-term aims, such as finding pride and excitement in academic pursuits. More value may be gained when shifting the focus from global adjustment to task-specific experiences.

### 4.4. Bifactor Indices and Need for Refined Narrow Assessments

The current study is the first to fully evaluate the bifactor implications for the psychometric properties of scoring and interpreting EI subscales. This carries implications for meta-analyses (Joseph and Newman 2010), conceptual models (Mayer et al. 2016), and individual reports which base conclusions on separate sub-branches without considering whether such scores make sense in the presence of a general factor. While our data suggest a certain degree of nonignorable multidimensionality within the GECo, the branches of understanding, perception, and emotion management may not hold enough unique reliable variance for robust research purposes and applications once accounting for general ability. This is concerning for diagnostic settings where educational and organizational interventions use the four-branch model of EI to craft separate modalities and feedback interventions geared towards specific skills (Brackett et al. 2012; Côté 2017). If using the GECo or MSCEIT, the low omega hierarchical values for specific EI branches are problematic because they imply large confidence intervals around a respondent’s scale score. Thus, interpretation of a person’s level of a specific EI ability will involve great uncertainty. In contrast, when scale scores are interpreted to represent a blend of general and specific ability constructs, the omega values of the scores are larger. Like bifactor conclusions from broader intelligence inventories (Styck 2019), modern EI instruments may only be equipped to answer the question, “How emotionally intelligent are you” rather than the specific question “In what ways are you emotionally intelligent.”

This unreliability carries forward to the uncertainty of factor score estimates and SEM models of EI branches, potentially explaining past empirical inconsistencies and their negligible incremental effects in the current investigation. While their latent representation is identified, the EI branches may not be reliably specified and hence could change meaning across studies even if configural structure remains intact. The S-1 bifactor model partially assuaged these concerns by improving secondary loadings on EI abilities but the construct reliability still fell short of the recommended .80 threshold (Rodriguez et al. 2016a). It should be noted that these issues are not isolated to the current study but afflict a majority of bifactor models in psychology (Table 1). Consequentially, empirical findings on EI branches may be unstable and lead to ambiguity on whether diverging effects arise from truly weak effects for an EI subcomponent or measurement artifacts. Relatedly, the incremental effects of emotion regulation may arise because it is a psychometrically superior stand-alone construct operating independent of general ability. It is possible that previous reviews overstate the role of emotion regulation skills because most tools are unable to fully capture the unique capacity to perceive and understand emotions (Joseph and Newman 2010).

One way to improve the situation is to expand the number and salience of narrow indicators underlying each EI branch. Theoretically, all the EI branches involve several related abilities, problem sets, and stimuli which could be used to expand the variety and scope of items for perceiving, understanding, and managing emotions (Mayer et al. 2016). For instance, the emotion perception branch includes the ability (a) to identify emotions in external stimuli, (b) to identify one’s own emotions, (c) to accurately express one’s own emotions, (d) to distinguish between genuine and feigned emotional expressions, and (e) to know displays rules for emotions expressed in different cultures (Mayer et al. 2016). However, in most EI research, including the present study, this branch is operationalized solely as the first ability. As such, there is opportunity to expand the precision of each EI branch using multiple sub-tasks to define and parse the effects of general ability from specific emotional skills.

### 4.5. Limitations and Future Directions

While the present study highlights new horizons for comprehensive EI batteries, several limitations should be noted. First, current findings do not model the full CHC hierarchy which necessitates numerous primary ability assessments covering multiple second-stratum abilities. MacCann et al. (2014) included 21 assessments capturing 5 s-stratum abilities, although there are plausibly upwards of 20 broad cognitive abilities (Schneider and McGrew 2018). While we expect current findings to generalize to other cognitive tests, loading patterns with overall ability may weaken or strengthen depending on whether overall ability is defined more by fluid relative to crystallized components (Völker 2020). Second, results are not directly contrasted with other EI batteries (e.g., MSCEIT) or stand-alone tests (e.g., Situational Tests of Emotional Understanding and Management (MacCann and Roberts 2008). The GECo and MSCEIT have been compared in predictive terms (Schlegel and Mortillaro 2019), but have not been jointly modeled in structural solutions. Future hierarchical models spanning multiple EI tests would provide converging evidence for EI constructs across batteries, scoring approaches, and modalities. We also were unable to model several criteria at varying levels of abstraction to fully test the compatibility principle. This endeavor would necessitate a hierarchical representation of performance criteria in education. Perhaps evaluating GPA by major and course-specific performance would allow the examination of whether EI branches better predict performance in humanities or theatrical courses (MacCann et al. 2020). Alternatively, expanded well-being measures can be used to model two bifactor solutions, in which overall EI predicts general well-being and EI branches predict well-being facets, such as positive relationships, growth, and mastery/acceptance (Chen et al. 2013). Finally, the sample was predominantly young females which may restrict generalizability. Age and sex are associated with EI ability scores (Cabello et al. 2016; Joseph and Newman 2010), suggesting the current sample may restrict the range of EI’s predictive potential. Future GECo investigations should diversify sex and age composition to determine if findings replicate in more varied samples.

As is evident from the limitations, the discovery of the appropriate breadth, number, and value of primary emotional abilities will be aided by an increasingly global strategy which accumulates research using common principles and variables derived from multiple streams on emotional competence. With the increasing number of EI tests and newly proposed emotional abilities, a fruitful next step is to follow the CHC tradition and begin mapping an inclusive hierarchy of EI tests. At the lowest realm will be elementary abilities embedded in specific tasks, such as defining the meaning of emotional words, categorizing feelings into broader families, or correctly identifying which events precede which feelings. In the middle are broader groupings of mutually correlated elementary abilities. For example, skill in defining, categorizing, and tracing emotions may cluster into emotion understanding. Sitting at the apex will be an overall EI factor indexing abstract reasoning about emotions. Multiple authors have proposed what the narrow EI indicators and alternative broader structures might look like (Castro et al. 2016; Mayer et al. 2016; Schneider et al. 2016).

Further, exploration of new EI tests may reveal deficiencies or omissions in the three-branch model. Incorporating newly proposed EI abilities, such as attentional regulation (Elfenbein et al. 2017) and expressive influence (Côté and Hideg 2011), with emotional information processing (Austin 2010; Fiori 2009) and attentional biases (Lea et al. 2018), might produce a more complex EI structure. For instance, Schlegel et al. (2012) found emotional perception tests are moderately fit by a general perception factor with nested minor skills marked by paired emotions existing in the same family (e.g., anger and irritation). Findings of loosely organized abilities may call for a reorganization of EI tasks into pure cognitive operations to resemble the CHC hierarchy such as breaking emotional perception into overlapping skills across visual processing and domain knowledge for certain associations. Alternatively, joint modeling of multiple EI batteries may support overlapping but distinguishable process-oriented models of intelligence by staging tasks as capturing different phases of information processing (Schneider and McGrew 2018). Either way, understanding the nature of EI could be accelerated by synthetic factor analytic efforts which bring new and old measures together under a single tent to produce a common nomenclature and framework for guiding future efforts.

Expanding further, researchers can investigate multiple EI tests in tandem with a wider array of “cold” cognitive abilities capturing impersonal knowledge and reasoning alongside newly proposed “hot” intelligences based on understanding other’s personalities and rules guiding social interactions (Schneider et al. 2016). Such efforts will not only clarify how EI fits within the pantheon of human abilities but may expand CHC to consider new aptitudes for performance critical to different domains of psychological functioning. However, future research needs to model a full array of broad CHC abilities such as fluid reasoning, verbal comprehension, and processing speed to evaluate if new theoretical abilities explain empirical findings consistent with known facts from cognitive, developmental, and biological psychology. Are these hot intelligences predominantly aptitudes swaying the general breadth and rate of learning new information or unique knowledge sets attained through culture and experience? Do they overlap in development and degradation at the same rate as fluid and crystallized cognitive abilities or remain impervious to the passage of time? Are hot intelligences just another form of semantic reasoning which fail to predict meaningful outcomes after controlling for the effects of verbal intelligence? These and numerous other questions necessitate inclusion of a fuller array of both hot and cool intelligences to convincingly show the CHC should be amended with new abilities (McGrew 2009; Schneider et al. 2016).

## 5. Conclusions

Based on multidimensional theories of intelligence, the current study supports the situation of the GECo’s emotion perception, understanding, and management branches within a hierarchical as well as oblique model of cognitive ability. Bifactor models and indices suggest existence of a general ability pervading performance across most EI branches but also the existence of EI sub-skills showing different ways of being emotionally intelligent. Results point to emotion regulation as distinct from general ability and personality, possibly existing in a domain of self-oriented skills. Moreover, emotion perception and regulation predict GPA and affective criteria beyond general fluid intelligence and personality with greater unique potential of EI related to emotional immersion in tasks.

Despite multidimensionality, precise estimation and modeling of several EI sub-skills appears problematic so primary interpretive emphasis for current EI assessments should be placed on the overall ability and emotional regulation scores (as operationalized by the GECo). If going beyond general ability to interpret EI sub-scale scores, researchers and practitioners must exercise caution to guard against overinterpretation of scores due to the indeterminate and low stability. This finding likely extends to other EI batteries such as the MSCEIT, thus necessitating more efforts to evaluate, develop, and refine specialized EI tasks to better isolate unique skills in perceiving, understanding, and managing others’ emotions.

## Figures and Tables

**Table 1:** Estimated 33rd and 66th percentiles for bifactor indices using Rodriguez et al. (2016b) Table 2 results.

Statistical Index	33rd Percentile	66th Percentile
*Omega* (total scale)	.92	.95
*Omega* (subscale)	.82	.90
*OmegaH*	.76	.84
*OmegaHS*	.20	.34
*ECV*	.61	.70
*PUC*	.63	.72
*FD* (general)	.93	.96
*FD* (group)	.76	.85
*H* (general)	.90	.93
*H* (group)	.48	.63

**Table 2:** Means, standard deviations, and correlations (*N* = 821).

Var	*M*	*SD*	1	2	3	4	5	6	7	8	9	10	11	12	13	14	15
ARM	3.58	1.87	.61														
ERA	.56	.12	.33 **	.66													
EU	.66	.13	.29 **	.34 **	.55												
ER	.56	.11	−.03	.00	−.01	.75											
EM	.44	.16	.26 **	.34 **	.35 **	.15 **	.61										
GECo	.55	.08	.35 **	.65 **	.68 **	.39 **	.79 **	.79									
O	4.98	1.04	.12 **	.13 **	.09 **	.11 **	.10 **	.17 **	.76								
C	4.65	1.17	−.07 *	−.14 **	−.07	.17 **	−.02	−.03	−.01	.78							
E	4.13	1.32	−.13 **	−.06	−.10 **	.15 **	−.07 *	−.05	.18 **	.03	.84						
A	5.48	.95	−.01	.11 **	.13 **	.06	.13 **	.17 **	.24 **	.03	.22 **	.77					
N	4.17	1.10	.05	.10 **	.03	−.30 **	−.02	−.06	−.06	−.19 **	−.11 **	.00	.73				
Thri	5.54	.98	−.13 **	−.12 **	−.07	.26 **	−.02	.00	.10 **	.27 **	.34 **	.20 **	−.32 **	.96			
PF	4.97	.92	−.13 **	−.10 **	−.04	.21 **	−.03	.00	.07 *	.15 **	.31 **	.20 **	−.29 **	.61 **	.92		
NF	3.39	1.08	.08 *	.11 **	.05	−.24 **	−.00	−.02	−.04	−.23 **	−.18 **	−.11 **	.44 **	−.52 **	−.53 **	.90	
AfE	5.30	1.02	−.05	−.06	.03	.17 **	.03	.06	.11 **	.13 **	.13 **	.20 **	−.19 **	.47 **	.52 **	−.38 **	.95
GPA	3.22	.51	.11 **	.19 **	.16 **	−.05	.11 **	.17 **	.10 **	.10 **	.05	.11 **	.04	.17**	.11 **	−.10 **	.08 *

Note. *M* and *SD* are used to represent mean and standard deviation, respectively. Cronbach alpha coefficients provided in diagonals. ARM = Raven’s Advanced Progressive Matrices—Short Form; ERA = Emotion recognition ability; EU = Emotion understanding; ER = Emotion regulation; EM = Emotion management; GECo = Total score for Geneva Emotional Competence Test; O = Openness to Experience; C = Conscientiousness; E = Extraversion; A = Agreeableness; N = Neuroticism; PF = Positive Feeling; NF = Negative Feeling; Afe = Affective Engagement; GPA = Cumulative grade point average. * indicates *p* < .05. ** indicates *p* < .01.

**Table 3:** Fit of the Four Factor Models to the GECo Branches and Raven’s Advanced Progressive Matrices-Short Form.

Model	$\chi^{2}$	*df*	TLI	CFI	RMSEA (90% CI)	SRMR	AIC	BIC
Model 1: One-factor	894.262	135	.546	.599	.083 (.078–.088)	.078	−6079.704	−5910.125
Model 2: Five-factor	117.990	125	1.00	1.00	.000 (.000–.015)	.025	−6842.713	−6626.029
Model 3: Hierarchical	146.341	130	.990	.991	.012 (.000–.022)	.033	−6824.688	−6631.556
Model 4: Bifactor	125.545	117	.994	.996	.009 (.000–.020)	.030	−6819.490	−6565.122
Model 5: Bifactor-mod	100.662	116	1.00	1.00	.000 (.000–.011)	.021	−6842.358	−6583.279

Note. GECo = Geneva Emotional Competence Test; TLI = Tucker-Lewis Index; CFI = comparative fit index; RMSEA = root mean square of approximation; SRMR = standardized root mean square residuals; AIC = Akaike information criterion; BIC = Bayesian information criterion’ Bifactor-mode = Bifactor model with residual correlation between emotional regulation and emotional management.

**Table 4:** Standardized Parameter Estimates (and Standard Error in Parentheses) from the Bifactor Model and Explained Common Variance (ECV), Factor Determinacy (FD), Construct Replicability (H), and Model-Based Reliability Indices.

Parcels	General	GF	ERA	EU	ER	EM
ARM1	.345 (.012)	.486 (.016)				
ARM2	.466 (.013)	.486 (.021)				
ARM3	.262 (.009)	.365 (.011)				
ERA1	.469 (.006)		.376 (.010)			
ERA2	.400 (.010)		.298 (.015)			
ERA3	.345 (.008)		.404 (.014)			
ERA4	.321 (.011)		.151 (.015)			
EU1	.329 (.009)			.419 (.022)		
EU2	.357 (.009)			.252 (.016)		
EU3	.318 (.011)			.231 (.020)		
EU4	.499 (.010)			.186 (.018)		
ER1	**.009 (.005)**				.617 (.005)	
ER2	**−.006 (.005)**				.600 (.005)	
ER3	.122 (.006)				.823 (.006)	
EM1	.499 (.011)					.422 (.015)
EM2	.333 (.010)					.399 (.014)
EM3	.296 (.009)					.272 (.013)
EM4	.416 (.010)					.398 (.015)
ECV_GS	.40	.40	.59	.64	.01	.52
ECV_SG	.40	.11	.07	.06	.25	.10
FD	.82	.66	.56	.51	.88	.62
H	.73	.44	.32	.27	.77	.40
$\omega \;\left(\omega_{S}\right)$	.78	.60	.56	.53	.73	.62
$\omega_{H}\left(\omega_{HS}\right)$	.59	.36	.22	.18	.77	.40

Note. ARM = Raven’s Advanced Progressive Matrices—Short Form; GF= Fluid intelligence; ERA = Emotion recognition ability; EU = Emotion understanding; ER = Emotion regulation; EM = Emotion management; ECV_GS = explained common variance of general factor with respect to specific factor, within-domain ECV; ECV_SG = explained common variance of a specific factor with respect to the general factor; specific-dimension ECV; FD = Factor determinacy; H = Construct replicability; ω = omega; ω_S_ = omega subscale; ω_H_ = omega hierarchical; ω_HS_ = omega hierarchical subscale. **Bolded** estimates non-significant at the .05 level.

**Table 5:** Multivariate Latent Regression analyses of the Bifactor (S-1) Model (Reference Facet = Fluid Intelligence) with GECo Branches and Big Five as Covaried Predictors and GPA, Well-Being Factors, and Affective Engagement as Covaried Outcomes.

	Correlations									Outcomes (Standardized Beta Weights)				
Factor	1	2	3	4	5	6	7	8	9	GPA	TH	PF	NF	AE
GA-FI										.138 **	−.108 **	−.125 **	.090 *	−.039
ERA	.00									.185 *	−.041	−.046	.018	−.158 *
EU	.00	.49 **								.115	.029	.038	.029	.131
ER	.00	.03	.03							−.129 *	.091 ^†^	.075	−.033	.115 *
EM	.00	.43 **	.53 **	.26 **						−.021	−.034	−.011	−.005	−.024
O	.00	.19 **	.12 ^†^	.16 **	.14 *					.043	−.056	−.102 *	.117 *	.026
C	.00	−.21 **	−.09	.23 **	.02	.02				.178 **	.189 **	.040	−.099 *	.105 *
E	.00	−.03	−.11 ^†^	.18 **	−.07	.26 **	.00			.083 ^†^	.295 **	.272 **	−.113 *	.056
A	.00	.18 **	.22 **	.07	.24 **	.36 **	.04	.26 **		.052	.169 **	.198 **	−.122 *	.214 **
N	.00	.14 *	.03	−.43 **	−.11 ^†^	−.18 **	−.34 **	−.17 **	.02	−.002	−.263 **	−.288 **	.543 **	−.117 *
*R2*										.119	.351	.282	.401	.162

Note. GA-FI ref = fluid intelligence reference factor for general ability; ERA = Emotion recognition ability; EU = Emotion understanding; ER = Emotion regulation; EM = Emotion management; O = Openness to Experience; C = Conscientiousness; E = Extraversion; A = Agreeableness; N =Neuroticism; GPA = Grade point average; TH = Thriving; PF = Positive feeling; NF = Negative feeling; AE = Affective Engagement. Coefficient of determination (*R*^2^). ** *p* < .01, * *p* < .05, ^†^ < .10.

## Data Availability

The data presented in this study are available on request from the corresponding author. The data are not publicly available due to confidential participant information.

## Footnotes

1 Technically Spearman proposed a two-factor theory of intelligence wherein each ability task is uniquely influenced by a second factor orthogonal to *g*. However, Spearman considered such factors largely nuisance and focused predominantly on a general ability.

2 We ran supplementary analyses applying a correlational parceling strategies (Landis et al. 2000), in which items were assigned in triplets based upon strongest associations. This expanded the number of factor indicators to 14 ERA parcels, 6 EU parcels, 9 ER parcels, and 7 EM parcels. Conclusions about model-quality and bifactor indices were the same; therefore, we retain the parsimonious facet-representative parceling strategy. Results for correlation parceling available upon request.

3 The reverse ordering is possible as well: people who are not doing so great at life may try to fix it by better focusing on improving skills in reading others’ emotions.

## Supplementary Materials

The following are available online at https://www.mdpi.com/2079-3200/9/1/14/s1, Table S1: Descriptive Statistics and Correlations between Parcels, Table S2: SEM Coefficients Regressing GECo Branches on APM and the Big Five.
