# Supplementary material for: How Multidimensional Is Emotional Intelligence? Bifactor Modeling of Global and Broad Emotional Abilities of the Geneva Emotional Competence Test
Source: J Intell. 2021 Mar 5;9(1):14. doi: 10.3390/jintelligence9010014 (PMC8006218; doi:10.3390/jintelligence9010014)
Supplement: Supplementary file 1 [file jintelligence-09-00014-s001.pdf]

Supplementary Materials and Tables

Table S1. Descriptive Statistics and Correlations between Parcels.

Variable	*M*	*SD*	1	2	3	4	5	6	7	8	9	10	11	12	13	14	15	16	17	18	19	20	21	22	23	24	25	26	27	28	29	30
ARM1	.36	.27																														
ARM2	.51	.34	.40																													
ARM3	.33	.21	.27	.30																												
ERA1	.50	.14	.18	.29	.12																											
ERA2	.56	.21	.14	.17	.11	.31																										
ERA3	.72	.18	.11	.19	.09	.31	.26																									
ERA4	.45	.25	.15	.13	.10	.21	.14	.19																								
EU1	.64	.18	.10	.15	.04	.12	.13	.09	.11																							
EU2	.62	.19	.13	.15	.10	.17	.13	.13	.09	.23																						
EU3	.69	.25	.10	.14	.11	.17	.09	.14	.12	.21	.14																					
EU4	.70	.23	.19	.22	.13	.22	.20	.16	.16	.22	.25	.22																				
ER1	.51	.12	−.08	−.03	.01	−.02	−.00	−.00	.01	.02	−.04	.02	−.04																			
ER2	.56	.13	−.06	−.03	−.08	−.00	−.02	.01	−.04	−.03	−.05	−.02	−.00	.37																		
ER3	.59	.15	−.01	.06	−.00	.03	.03	.02	−.02	.03	−.01	−.01	.06	.51	.49																	
EM1	.55	.27	.11	.20	.13	.20	.23	.15	.16	.21	.22	.15	.27	.08	.07	.16																
EM2	.41	.23	.14	.16	.11	.13	.13	.12	.09	.11	.12	.07	.17	.09	.07	.14	.34															
EM3	.36	.22	.14	.08	.06	.12	.17	.10	.12	.14	.09	.13	.13	.08	.01	.07	.25	.21														
EM4	.42	.24	.13	.20	.08	.19	.18	.15	.16	.14	.13	.15	.19	.05	.07	.10	.38	.29	.25													
PF1	5.15	1.02	−.13	−.05	−.05	−.04	−.06	−.06	−.10	−.00	.02	−.03	−.08	.17	.18	.17	.02	.01	−.08	.05												
PF2	5.05	1.02	−.17	−.09	−.10	−.04	−.08	−.08	−.10	−.01	−.02	−.02	−.06	.17	.15	.13	−.01	−.03	−.06	.03	.78											
PF3	4.72	1.03	−.16	−.02	−.09	−.04	−.03	−.08	−.04	.01	−.02	−.03	−.05	.16	.15	.12	−.00	−.07	−.13	.03	.66	.71										
NF1	3.29	1.25	.13	.13	.07	.12	.06	.06	.14	−.01	.04	.06	.08	−.20	−.17	−.20	.01	−.02	.09	.03	−.53	−.52	−.39									
NF2	3.48	1.21	.11	.03	.04	.06	.07	.02	.13	−.03	.04	.07	.08	−.16	−.18	−.18	−.02	−.03	.07	.01	−.47	−.48	−.36	.74								
NF3	3.40	1.21	.00	−.02	−.01	.02	.07	−.01	.03	−.03	.00	−.01	−.00	−.11	−.18	−.17	−.10	−.02	.03	−.06	−.38	−.38	−.26	.59	.64							
THR1	5.25	1.16	−.13	−.11	−.05	−.10	−.10	−.06	−.14	−.00	−.04	−.02	−.11	.21	.21	.18	−.04	−.00	−.04	−.05	.58	.56	.48	−.51	−.51	−.34						
THR2	5.54	1.05	−.10	−.09	−.06	−.07	−.06	−.04	−.12	−.02	−.02	−.03	−.10	.20	.22	.20	−.04	.02	−.02	−.02	.53	.51	.44	−.44	−.48	−.32	.81					
THR3	5.92	0.90	−.11	−.06	−.07	−.02	−.00	−.02	−.11	.02	−.02	−.02	−.05	.21	.20	.16	.02	.04	−.00	.00	.52	.49	.40	−.43	−.45	−.31	.80	.77				
AFE1	5.78	0.92	−.06	−.04	−.01	−.05	−.04	−.04	−.07	.01	.04	−.01	.01	.10	.11	.15	.05	.03	−.03	.01	.51	.45	.37	−.34	−.31	−.24	.45	.47	.43			
AFE2	4.90	1.09	.01	.00	.00	.01	−.02	−.02	−.05	−.01	.05	.02	.06	.10	.12	.19	.12	.05	−.01	.02	.42	.37	.32	−.31	−.30	−.30	.37	.41	.34	.71		
AFE3	5.40	1.23	−.05	−.06	−.06	−.02	−.04	−.04	−.06	.04	.04	.00	−.01	.09	.06	.13	.05	−.01	−.00	−.03	.48	.47	.39	−.35	−.32	−.25	.42	.40	.36	.77	.65	
GPA	3.22	0.51	.07	.09	.08	.14	.13	.14	.09	.06	.10	.16	.11	−.04	−.03	−.05	.12	−.01	.10	.09	.10	.12	.09	−.07	−.12	−.08	.16	.15	.17	.11	.07	.08

Table S2. SEM Coefficients Regressing GECo Branches on APM and the Big Five.

Factor	Standardized Beta Weights			
	ERA	EU	ER	EM
GF	.52**	.47*	.03	.41**
O	.10	.03	.06	.02
C	−.14*	−.10	.10*	−.03
E	−.04	−.15	.09	−.13*
A	.14*	.24**	.04	.26**
N	.08	−.05	−.36*	−.13*
R	.61	.56	.45	.50
R^2	.37	.32	.21	.25

Note. GF = Fluid intelligence; O = Openness to Experience; C = Conscientiousness; E = Extraversion; A = Agreeableness; N = Neuroticism; ERA = Emotion recognition ability; EU = Emotion understanding; ER = Emotion regulation; EM = Emotion management. * indicates $p < .05$. ** indicates $p < .01$.

Table S3. Commonality Analyses on EI Scales and GPA.

Predictor Subset	Coefficient	% Total
RMscore	0.001	0.017
EM_score	0.001	0.017
ERA_score	0.014	0.285
EU_score	0.007	0.138
EReg_score	0.003	0.059
RMscore,EM_score	0	0.005
RMscore,ERA_score	0.002	0.046
EM_score,ERA_score	0.002	0.046
RMscore,EU_score	0.001	0.02
EM_score,EU_score	0.002	0.035
ERA_score,EU_score	0.005	0.103
RMscore,EReg_score	0	0.004
EM_score,EReg_score	0	−0.009
ERA_score,EReg_score	0	0.006
EU_score,EReg_score	0	0.01
RMscore,EM_score,ERA_score	0.001	0.024
RMscore,EM_score,EU_score	0.001	0.014
RMscore,ERA_score,EU_score	0.002	0.05
EM_score,ERA_score,EU_score	0.004	0.077
RMscore,EM_score,EReg_score	0	−0.002
RMscore,ERA_score,EReg_score	0	0.004
EM_score,ERA_score,EReg_score	−0.001	−0.01
RMscore,EU_score,EReg_score	0	0.003
EM_score,EU_score,EReg_score	0	−0.008
ERA_score,EU_score,EReg_score	0	0.005
RMscore,EM_score,ERA_score,EU_score	0.004	0.071
RMscore,EM_score,ERA_score,EReg_score	0	−0.003
RMscore,EM_score,EU_score,EReg_score	0	−0.002
RMscore,ERA_score,EU_score,EReg_score	0	0.005
EM_score,ERA_score,EU_score,EReg_score	0	−0.006
RMscore,EM_score,ERA_score,EU_score,EReg_score	0	−0.004

To check for possible suppression we ran a commonality analysis with GPA as outcome and fluid intelligence along with EI branches as predictors. Commonality analysis performs all possible subsets regression for a set of predictors to break down effects into unique and common variance across all possible sets. Negative commonality coefficients large in magnitude indicate predictors which are affecting each other in opposing directions or that one variable confound the predictive power of another. This is suggestive of suppression but not definitive because negative coefficients can arise when predictors have opposing correlations.

Above is a table of the results which show mostly positive to small or nill common effects. All pairings with ER show some signs of small negative commonalities but their coefficients are often zero indicating ER does not help other predictors gain greater weight in a regression equation. Further, to the extent ER suppresses variance it would only account for less then .01% of the overall R-squared for GPA (sum of % total of negative effects * by overall R-squared).

We also ran a multiple regression to identify if the signs of any predictor beta weights switched or strengthened compared to their bivariate counterparts and found no such exaggerations. Hence, it appears ER just seem to have little to no relationship with GPA in the current findings and does not appear to increase the predictive power of the other predictor's unique effects.
